# Storming the barricades of rhamnogalacturonan-II synthesis and function

**DOI:** 10.1093/plcell/koaf088

**Published:** 2025-04-16

**Authors:** Quentin Hays, Patrice Lerouge, Marc Ropitaux, Charles T Anderson, Arnaud Lehner

**Affiliations:** Université de Rouen Normandie, GLYCOMEV UR 4358, SFR Normandie Végétal FED 4277, Innovation Chimie Carnot, IRIB, F-76000 Rouen, France; Department of Biology, The Pennsylvania State University, University Park, PA 16802, USA; Université de Rouen Normandie, GLYCOMEV UR 4358, SFR Normandie Végétal FED 4277, Innovation Chimie Carnot, IRIB, F-76000 Rouen, France; Université de Rouen Normandie, GLYCOMEV UR 4358, SFR Normandie Végétal FED 4277, Innovation Chimie Carnot, IRIB, F-76000 Rouen, France; Department of Biology, The Pennsylvania State University, University Park, PA 16802, USA; Université de Rouen Normandie, GLYCOMEV UR 4358, SFR Normandie Végétal FED 4277, Innovation Chimie Carnot, IRIB, F-76000 Rouen, France

## Abstract

Despite its low abundance, rhamnogalacturonan-II (RG-II) is an essential structural component of the cell wall and is present in a highly conserved molecular configuration across all plants. RG-II is a branched pectin domain that contains 13 different sugars linked by over 20 different bond types, and uniquely among pectins it can be covalently dimerized via borate diesters. RG-II is hypothesized to crosslink the pectin matrix, controlling cell wall architecture and porosity, but has resisted detailed analyses due to its compositional complexity and the lethality of RG-II-deficient mutants. Here, we highlight how biochemical dissection, genetic engineering, chemical inhibitors, and high-resolution imaging have enabled recent leaps in our understanding of RG-II structure, synthesis, localization, dimerization, and function, pointing out new questions and research directions that have been enabled by these advances.

## Teaser

This review highlights breakthroughs in understanding rhamnogalacturonan-II structure, synthesis, localization, and function in plants and proposes future research into this enigmatic polymer.

## Introduction

One unique attribute of plant cells is the presence of a cell wall. The cell wall is an extracellular matrix that regulates plant growth and development (Cosgrove 2016; Zhang et al. 2021). It is also the first line of defense against pathogens and abiotic stresses (Bacete et al. 2018; Anderson and Kieber 2020). To meet societal needs, increasing understanding of the cell wall is essential to improve our capacity to grow plants, protect them against stressors, and extract and convert plant biomass more efficiently to produce bioenergy and bioproducts (Marriott et al. 2016; Devree et al. 2021; Tingley et al. 2021).

The plant cell wall is an extracellular layer with a complex organization of polysaccharides, proteins, and phenolic compounds. Polysaccharides, the predominant element in the cell wall, are composed of 3 families of glycans: cellulose, hemicelluloses, and pectins. Cellulose is a linear polymer of β-1,4–linked glucan found in the cell wall as microfibrils formed by the association of 18 to 24 individual chains (Anderson and Kieber 2020). Hemicelluloses often have a β-1,4-glucan or xylan backbone with a large variety of side chains (Scheller and Ulvskov 2010). Finally, pectins are mainly represented by homogalacturonans (HGs), rhamnogalacturonan I (RG-I), and rhamnogalacturonan II (RG-II). HGs are linear α-1,4-galacturonic acid chains that are partially methylesterified or *O*-acetylated (Anderson and Kieber 2020). When they are de-methylesterified by pectin methylesterases (PMEs), HGs can interact with Ca^2+^ ions, forming an egg box structure leading to stiffer walls and cell adhesion, or be cleaved by polygalacturonases (PGs) and pectate-like lyases (PLLs), inducing cell wall loosening (Wolf et al. 2009; Sénéchal et al. 2014; Anderson and Kieber 2020; Temple et al. 2022). In contrast, RG-I domains have a backbone made of repeating units of α-1,4 galacturonic acid and α-1,2 rhamnose with arabinans, galactans, and arabinogalactans side chains (Kaczmarska et al. 2022). Finally, RG-II is composed of an HG backbone substituted by 6 side-chains and so, RG-II can be considered as a third pectic domain or a subdomain of HG. Although representing 0.5% to 5% of the dry mass of the cell wall, RG-II has specific features that confers to this 5-kDa glycan a unique position in the understanding of the role of the plant cell walls. RG-II motif is composed of 13 different sugars, some of which are found only in RG-II, linked by over 20 different bond types into a highly complex oligomer that is conserved in the plant kingdom and that likely appears during their terrestrialization (Darvill et al. 1978; Matsunaga et al. 2004; Barnes et al. 2021; Lerouge et al. 2021).

Moreover, RG-II is a unique pectic domain found mostly as a dimer covalently linked through a borate diester bond (Funakawa and Miwa 2015). Despite its low abundance in the wall, defects in RG-II synthesis or dimerization lead to severe phenotypes or lethality (O'Neill et al. 2001; Miwa et al. 2013; Smyth et al. 2013; Dumont et al. 2014, 2015; Zhao et al. 2020; Peng et al. 2021; Zhang et al. 2024). Altogether, the proportions, interactions, and modifications of polysaccharides determine the mechanical properties of the cell wall and its capacity to expand (Anderson and Kieber 2020). Cellulose and hemicelluloses together are often described as the main players in cell wall strengthening and expansion (Scheller and Ulvskov 2010; Xiao et al. 2016; Cosgrove 2024). However, the importance of the cellulose/hemicellulose network in cell wall growth is debated because of the absence of major defects in plants lacking xyloglucan (Cavalier et al. 2008; Sowinski et al. 2022). Compensation by galactoglucomannan, the content of which is increased in these xyloglucan-deficient mutants, might explain the lack of growth impairment in these lines (Yu et al. 2022). Nevertheless, the roles of pectic polysaccharides in determining the properties of the cell wall are underappreciated compared with the severe phenotypes displayed in their mutant lines (Dumont et al. 2015; Du et al. 2020, 2022; Zhao et al. 2020). Thus, although knowledge of the biochemical structure of RG-II has steadily increased over the past several decades, insights into its biosynthesis, mechanism of dimerization, localization, and function have been difficult to uncover. Here, we highlight several recent breakthroughs in our understanding of RG-II synthesis, structure, and function that are based on cutting-edge combinations of enzymatic deconstruction and structural analysis, biochemical tools for studying RG-II dimerization in vivo, and novel genetic and cell biological approaches that bypass the lethality of RG-II defects. We also present several new hypotheses concerning RG-II synthesis, function, and evolution, including that RG-II is permanently dimerized in the walls of living cells after its synthesis; that despite having a backbone made of galacturonic acid, the side chains of RG-II protect this backbone from cleavage; and that phenotypes arising from RG-II defects mimic those arising from boron deprivation. Continuing to expand the toolkit of methods used to study RG-II will help elucidate the molecular mechanisms by which RG-II is built and dynamically shapes the cell wall during plant growth and development.

## Elucidating the complex structure of RG-II

### RG-II isolation and purification

**Figure 1.**
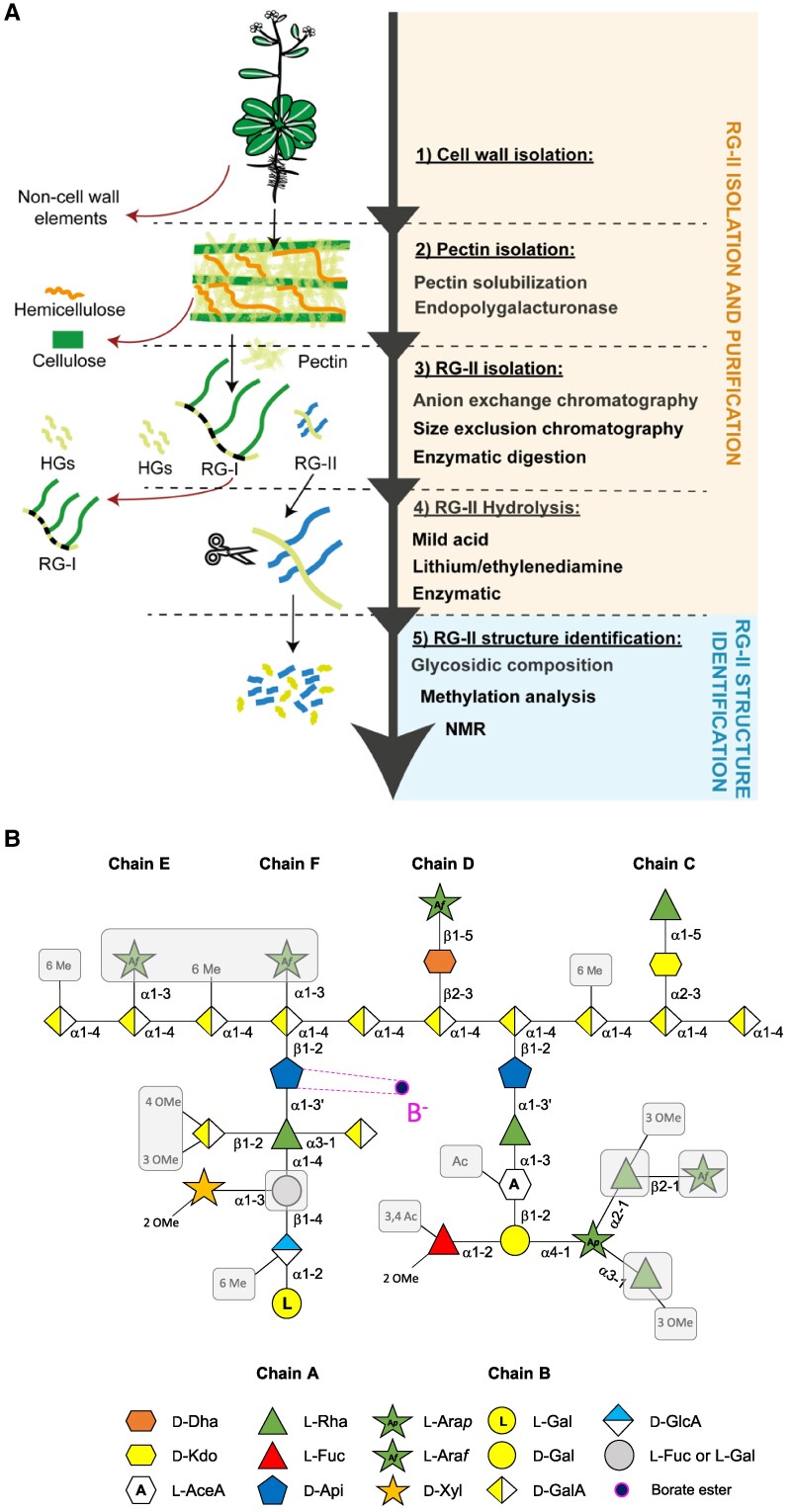
Schematic of the RG-II isolation from raw plant material to the identification of its composition and structure. **A)** Steps for isolating RG-II domain and determining its structure and composition. **B)** RG-II structure as resolved to date. The exact side chain positions have not yet been determined and are hypothetical in this figure. Monosaccharides, *O*-methyl groups, and acetyl groups with a gray background are found in RG-II from a subset of species.

As one of the most recently discovered pectin types, the structure of RG-II only started to come to light less than 50 years ago when Darvill et al. (1978) identified a complex minor polysaccharide in suspension-cultured sycamore cell walls. The first step in characterizing RG-II structure is refining cell walls from plant material (Fig. 1). The tissue can be flash frozen and ground in liquid nitrogen, yielding a fine homogenous powder that can be washed and centrifuged several times with 70% ethanol and/or a methanol-chloroform mix (1:1) before a final acetone wash and air drying (Chormova et al. 2014; Dumont et al. 2015). The alcohol insoluble residue obtained in this way contains the cell walls. This alcohol insoluble residue is saponified with Na_2_CO_3_ to de-methylesterify HGs and improve the later isolation of RG-II via endoPG treatment. Then the pH is neutralized before the solubilization of the pectin with ammonium oxalate, ammonium acetate, cyclohexanediaminetetraacetic acid, or EDTA (ethylenediaminetetraacetic acid), which interact with cations (Dumont et al. 2015; Barnes et al. 2021) (Fig. 1A). Even though cyclohexanediaminetetraacetic acid and EDTA can also solubilize pectin, ammonium oxalate can provide better results (Mort et al. 1991). To separate the different classes of pectin from each other, enzymatic digestion with endopolygalacturonase (endoPG) is used to cleave the HG backbone, freeing RG-I and RG-II from multi-domain pectic chains (Talmadge et al. 1973; Darvill et al. 1978; Chormova et al. 2014) (Fig. 1A). After this step, the soluble fraction is enriched in pectin and can be easily separated from the pellet containing the hemicellulose and cellulose fraction. Isolation of RG-II domain from RG-I and HGs can then be performed using 2 chromatography methodologies (Fig. 1A). Anion exchange chromatography enables the separation of pectic components according to their overall negative charge, whereas size exclusion chromatography (SEC) separates pectins based on molecular mass (Doco et al. 2001; Barnes et al. 2021). In this latter analytical strategy, the RG-II domain can be separated from larger RG-I and smaller oligogalacturonides. Moreover, separation of RG-II monomers and dimers can be efficiently achieved by SEC, as well as by gel electrophoresis (Chormova et al. 2014). Monosaccharide analysis of column fractions can be performed to check for RG-II purification (Ndeh et al. 2017; Barnes et al. 2021) (Fig. 1A).

### Glycosyl composition of RG-II

The first glycosyl compositional analysis of the neutral monosaccharides from RG-II was performed by gas chromatography coupled with mass spectrometry electron impact of alditol acetates (Darvill et al. 1978) (Fig. 1A). The presence of rhamnose, fucose, arabinose, galactose, glucose, and rarely found monosaccharides, such as apiose, 2-*O*-methyl-fucose, and 2-*O*-methyl-xylose, was detected by comparing their retention times to standards and quantified using standard monosaccharides (Darvill et al. 1978). As acidic glycosyl compounds could not be directly identified by analysis of alditol acetates, a colorimetric method was used, taking advantage of the capacity of uronic acids to form a specific chromogen in certain conditions, to detect the presence of uronic acids in RG-II (Blumenkrantz and Asboe-Hansen 1973; Biswal et al. 2017). These were further identified as galacturonic acid and glucuronic acid by reduction to 6,6-di-deutero hexoses followed by alditol acetate derivatization and mass spectrometry, indicating that RG-II is composed of 9 different monosaccharides (Darvill et al. 1978). A few years after the discovery of RG-II, aceric acid, a previously unreported monosaccharide, was identified thanks to ^1^H-NMR, ^13^C-NMR spectroscopy, and periodate oxidation degradation (Spellman et al. 1983; Pandeirada et al. 2022). Later, 2 additional monosaccharides were discovered: Kdo (3-deoxy-D-manno-2-octulosonic acid, York et al. 1985) and Dha (3-deoxy-D-lyxo-2-heptulosaric acid, (Stevenson et al. 1988), which are, to date, in plants found exclusively in RG-II, bringing the tally to 12 different monosaccharides. Currently, to determine the glycosyl composition of cell wall samples, methods other than alditol acetate derivatization are commonly used. Methanolysis coupled to trimethylsilyl derivatization for GC-MS analysis or high-performance anion-exchange chromatography of nonderived monomers are usually performed for the identification and quantification of both neutral and acidic monosaccharides (Doco et al. 2001; Biswal et al. 2017; Barnes et al. 2021). However, the lack of some available monosaccharide standards such as Dha or aceric acid makes their identification more difficult. Identification of these monomers can be ensured by coupling of GC-MS to electron impact mass spectrometry based on their electron impact fragmentation mass spectra (Doco et al. 2001). Together, these data have revealed the complex monosaccharide composition of RG-II.

### Linkages and structure of RG-II

The decoding of RG-II structure was first established through a partial deconstruction of RG-II by mild acid hydrolysis and lithium/ethylenediamine treatments, purification of the resulting RG-II fragments by filtration, and GC-MS analyses (Whitcombe et al. 1995; Edashige and Ishii 1998). An informative alternative was to use enzymes from fungi or bacteria to digest RG-II, breaking the glycosidic linkages (Vidal et al. 1999, 2000; Ndeh et al. 2017). The nature and the linkages of the isolated RG-II fragments were investigated by glycosyl composition methods (see above), methylation analysis to determine specific linkages, and NMR (Fig. 1A). The methylation analysis consists of the methylation of free hydroxyl group in an oligosaccharide followed by hydrolysis, reduction and acetylation (Sims et al. 2018). The acetylation occurs only in the hydroxyl groups that are not methylated, indicating the position(s) of the glycosidic bond(s) between monomers. The partially methylated alditol acetates (PMAA) obtained are analyzed in GC-MS-EI and compared with PMAA standards based on their specific retention time and fragmentation mass spectra (Sims et al. 2018). With PMAA, H-NMR and C-NMR and an original acidic depolymerization have helped to elucidate the nature of RG-II fragments (Pellerin et al. 1996; du Penhoat et al. 1999; Vidal et al. 2000; Glushka et al. 2003; Séveno et al. 2009; Pabst et al. 2013). All these methods together led to the identification of a backbone composed of a galacturonic acid chain 7 to 9 units long and a variety of side chain decorations ranging from an individual monosaccharide to an oligosaccharide composed of 9 units (Fig. 1B) (Darvill et al. 1978; Spellman et al. 1983; York et al. 1985; Stevenson et al. 1988; Puvanesarajah et al. 1991; Whitcombe et al. 1995; Pellerin et al. 1996; Edashige and Ishii 1998; Vidal et al. 2000; Glushka et al. 2003; Pérez et al. 2003; Séveno et al. 2009; Pabst et al. 2013; Ndeh et al. 2017). At present, the number of different side chains present on RG-II ranges from 4 to 6 (A-F) depending on the species and tissue (Lerouge et al. 2021) (Fig. 1B). Side chain A is an oligosaccharide of 8 monosaccharides well conserved except for substitution of L-Fuc by L-Gal in some species (Fig. 1B) (Pabst et al. 2013). Side chain B decorations can be more variable between species, containing 6 to 9 monosaccharides (Fig. 1B). Side chains C and D seem always to be disaccharides (Fig. 1B). Moreover, the backbone can be substituted by 2 α-L-arabinofuranosyl side chains at different positions (E and F) in RG-II from wine (Fig. 1B) (Pérez et al. 2003; Ndeh et al. 2017). Importantly, RG-II is mainly found as a dimer in the cell wall through a borate diester bond. Most of the boron in plant tissue has been detected in the plant cell wall fraction (Ishii and Matsunaga 1996; Matoh et al. 1996). ^11^B-NMR, ^1^H-NMR, ^13^C-NMR, and glycosidic linkages analyses established that this borate diester occurs between 2 apiose residues of the RG-II side chain A (Ishii and Matsunaga 1996; O’Neill et al. 1996; Pellerin et al. 1996; Ishii and Ono 1999; Ishii et al. 1999) (Fig. 1B). The polysaccharide can also carry either *O*-methylation or *O*-acetylation modifications on some residues from the side chains and backbones (Matsunaga et al. 2004; Ndeh et al. 2017; O’Neill et al. 2020) (Fig. 1B). Recently, it has been shown that the RG-II dimer is more extensively acetylated than monomers (O'Neill et al. 2020). Moreover, acetylation patterns on RG-II do not appear stochastic as shown in cell walls from plasmodesmata, suggesting that nonglycosidic residues influence RG-II biological function in the apoplast (O'Neill et al. 2020; Paterlini et al. 2022). The complexity of RG-II structure implies that plants deploy sophisticated cell machinery to allow its coordinated synthesis, as discussed below.

## Navigating the assembly line of RG-II biosynthesis

### Pectin synthesis

Pectic polysaccharide synthesis requires the activity of at least 67 distinct enzyme activities (glycosyltransferases, *O*-methyltransferases, *O*-acetyltransferases), catalyzing the transfer of, respectively, a sugar, a methyl or an acetyl group from a dinucleotide-sugar, S-adenosylmethionine, or acetyl CoA onto a carbohydrate acceptor (Atmodjo et al. 2013). The relevant glycosyltransferases are mainly transmembrane proteins localized in the Golgi apparatus cisternae (Kim and Brandizzi 2014). Moreover, these enzymes require pH to be maintained close to their optima in Golgi compartments to properly synthesize noncellulosic polysaccharides such as pectins (Onuh and Miwa 2023). After being synthesized in the Golgi apparatus, pectins are transported in vesicles to the plasma membrane and finally secreted to the cell wall (Mohnen 2008; Kim and Brandizzi 2014). It should be noted that, among pectins, the biosynthesis of RG-II domain also requires the cytosolic biosynthesis of certain specific nucleotide sugars, including UDP-β-L-Ara*p*; UDP-α-D-Xyl; UDP-α-D-Api*f*; GDP-β-L-Gal; CMP-β-D-Kdo and unknown D-Dha; and L-Ace*f*A (Atmodjo et al. 2013). The nucleotide forms of the last 2 sugars are undefined.

### RG-II backbone synthesis: similar to HGs?

Table 1 summarizes the glycosyltransferases involved in RG-II synthesis for which biochemical activity has been demonstrated or that are still putative and awaiting biochemical evidence, and Table 2 summarizes enzymes required for the synthesis of the monosaccharides present in RG-II (Figueroa et al. 2021). As for other pectic domain, RG-II is synthesized in the Golgi apparatus before being secreted to the cell wall. The first question that arises is the origin of its backbone, consisting of a small chain of 7 to 9 α-1-4–linked galacturonic acids, similar to HG. In the Golgi apparatus, HGs are synthesized by enzymes from the GT8 gene family, with putative or demonstrated galacturonosyltransferase (GAUT) activity, composed of 25 genes with 15 GAUTs and 10 GAUT-like genes (GATL) (Sterling et al. 2006; Caffall et al. 2009). T-DNA insertions in individual GAUT genes often do not result in phenotypes, emphasizing functional redundancy for galacturonosyltransferases (Caffall et al. 2009), as demonstrated where gaut5/gaut6/gaut7 (Lund et al. 2020) and gaut13/gaut14 (Wang et al. 2013) mutants show swollen pollen tubes and decreased pollen germination rates but single mutants do not. GAUT1, GAUT4, GAUT 10, GAUT11, GAUT13, and GAUT14 show validated galacturonosyltransferase activity, confirmed by expression in *Nicotiana benthamiana* cells or HEK293 cells in vitro (Sterling et al. 2006; Biswal et al. 2018; Voiniciuc et al. 2018; Engle et al. 2022). GAUT1, despite having galacturonosyltransferase activity, lacks the transmembrane domain required for integration into the Golgi membrane. Coimmunoprecipitation of GAUT1 with GAUT7 and GFP coexpressing lines have demonstrated that GAUT1:GAUT7 forms a heteromeric complex, with GAUT7 lacking catalytic activity but helping retain GAUT1 at the membrane via its transmembrane domain (Atmodjo et al. 2011). GAUT13 and GAUT14, at a high rate, and GAUT1:GAUT7 complex, at low rate, have the ability to initiate de novo synthesis of HGs (Atmodjo et al. 2011; Amos et al. 2018; Engle et al. 2022). GAUT4 knockdown (KD) plants display not only a reduction of HGs but also of RG-II as quantified by measuring the amount of 2-*O*-methyl-xylose, 2-*O*-methyl-fucose, and boron in pectin-enriched extracts (Biswal et al. 2018). In GAUT4-KD plants, both the reduction of HGs and RG-II seem to loosen the cell wall, increasing its porosity (Biswal et al. 2018). The involvement of GAUT4 in RG-II synthesis was also suggested through computational analyses (Voxeur et al. 2012). The differences in cell wall composition in the all T-DNA GAUT lines (Caffall et al. 2009) and the segregation of activity with de-novo synthesis and elongation of pre-existent HGs acceptor reveal a specificity of function for different acceptors among the GAUTs (Atmodjo et al. 2011; Amos et al. 2018; Engle et al. 2022). However, a GAUT that is specific for RG-II biosynthesis has not yet been isolated. GAUT4 seems to be the best candidate as a putative enzyme for RG-II backbone synthesis (Table 1). However further studies using in vitro assays or genetic manipulation are needed to test this hypothesis (Biswal et al. 2018).

**Table 1.** Summary of all the putative and confirmed (bold) glycosyltransferases involved in RG-II synthesis

Gene name/identifier	CAZY family	Function	Notes	Reference
**RG-II backbone**				
** GAUT4** At5g47780	**GT8**	**α-(1,4) galacturonosyltransferase**	**RGII synthesis, making GAUT4 the first HG:GalAT implicated in the reduction of both HG and RGII in the GAUT4-KD biomass suggests that GAUT4-synthesized HG is used as the backbone for RG-II synthesis.**	**Biswal et al. (2018) (*Panicum virgatum, Oryza sativa, Populus deltoides*)**
GAUT1 At3g61130	GT8	α-(1,4) galacturonosyltransferase	GalA transferase activity was proven for HG synthesis but no information regarding the synthesis of RG-II	Sterling et al. (2006) (*Arabidopsis thaliana*)
GAUT7 At2g38650	GT8	Putative α-(1,4) galacturonosyltransferase	No GalA Transferase activity proven. Involved in the formation of the complex with GAUT1	Atmodjo et al. (2011) and Lund et al. (2020) (*Arabidopsis thaliana*)
GAUT8/QUA1 At3g25140	GT8	Putative α-(1,4) galacturonosyltransferase	HG level is reduced in mutant lines but no information regarding RG-II	Bouton et al. (2002) (*Arabidopsis thaliana*)
RG-II side chains				
**RGXT1** **At4g01770**	**GT77**	**α-1,3-Xylosyltransferase**	**RG-II isolated from both *rgxt1* and *rgxt2* T-DNA insertional mutants functioned as specific acceptor molecules in the xylosyltransferase assay suggesting that single mutants contain complete and xylose-free RG-II**	**Egelund et al. (2006) (*Arabidopsis thaliana*)**
**RGXT2** **At4g01750**	**GT77**	**α-1,3-Xylosyltransferase**		**Egelund et al. (2006) (*Arabidopsis thaliana*)**
**RGXT3** **At1g56550**	**GT77**	**α-1,3-Xylosyltransferase**	**Recombinant protein was shown to catalyse transfer of D-xylose from UDP-α-D-xylose onto methyl α-L-fucoside**	**Egelund et al. (2008) (*Arabidopsis thaliana)***
**RGXT4/MGP4** **At4g01220**	**GT77**	**α-1,3-Xylosyltransferase**	**Recombinant protein exhibited xylosyltransferase activity and transferred d-xylose onto l-fucose. RG-II isolated from mutant exhibited a 30% reduction in 2-*O-*methyl D-xylose residues**	**Liu et al. (2011) *(Arabidopsis thaliana)***
**Cdi** **At1g64980**	**Putative GT8**	**GDP-L-galactose transferase**	**Biochemical and mass spectrometry assays showed that Cdi catalyzes the transfer of GDP-L-galactose to the terminus of side chain A on RG-II**	**Peng et al. (2021) (*Arabidopsis thaliana*)**
**MGP2/RCKT1** **At1g08660**	**GT29**	**RG-II CMP-Kdo transferase**	**Heterozygous *mgp2* mutant had a loss of male gametophytic function without affecting the female gametophyte. Analysis of rckt1 callus revealed a loss of 3-deoxy-D-manno-octulosonic acid (Kdo) from RG-II side chain C**	**Deng et al. (2010) and Zhang et al. (2024) (*Arabidopsis thaliana*)**
SIA2 At3g48820	GT29	Putative RG-II CMP-Kdo transferase	Heterozygous *sia2* mutant had a loss of male gametophytic function without affecting the female gametophyte	Dumont et al. (2014) (*Arabidopsis thaliana*)

**Table 2.** Summary of all the putative and confirmed enzymes involved in RG-II monosaccharides synthesis (Figueroa et al. 2021)

Gene name/identifier	Monosaccharide	Localization	Function	Reference
MUR1 At3g51160	GDP-L-Fuc	Cytosol	GDP-D-Mannose-4,6-dehydratase	O’Neill et al. (2001) and Pabst et al. (2013) (*Arabidopsis thaliana*)
GME At5g28840 Solyc01g097340 Solyc09g082990	GDP-L-Gal	Cytosol	GDP-D-Mannose -3,5-epimerase	Voxeur et al. (2011) and Qi et al. (2017) (*Solanum lycopersicum, Arabidopsis thaliana*)
AXS (1–2) At2g27860 At1g08200	UDP-D-Api UDP-D-Xyl	Cytosol	UDP-Api/UDP-Xyl synthases	Mølhøj et al., (2003), Ahn et al. (2006) and Zhao et al. (2020) (*Arabidopsis thaliana*)
CKS	CMP-Kdo	Mitochondria	CMP-Kdo synthetase	Kobayashi et al. (2011) (*Arabidopsis thaliana*)
UXS (1–5) At3g53520 At3g62830 At5g59290 At2g47650 At3g46440	UDP-D-Xyl	Cytosol/Golgi	UDP-Xyl synthase	Harper and Bar-Peled (2002) (*Arabidopsis thaliana*)
RHM (1–3) At1g78570 At1g53500 At3g14790	UDP-L-Rha	Cytosol	UDP-D-glucose 4,6-dehydratase, UDP-4-keto-6-deoxy-D-glucose 3,5-epimerase UDP-4-keto- L -rhamnose 4-keto-reductase	Oka et al. (2007) (*Arabidopsis thaliana*)
MUR4 At1g30620	UDP-L-Ara*p*	Golgi	UDP-Xyl 4-epimerase	Burget et al. (2003) (*Arabidopsis thaliana*)
RGP (1–3) At3g02230 At5g15650 At3g08900	UDP-L-Ara*f*	Cytosol	UDP-Ara mutase	Rautengarten et al. (2011) (*Arabidopsis thaliana*)
UGE (1–5) AT1G12780 AT4G23920 AT1G63180 AT1G64440 AT4G10960 USPase	UDP-D-Gal	Cytosol/Golgi	UDP-Glc 4-epimerase	Rösti et al. (2007) and Decker and Kleczkowski (2019) (*Arabidopsis thaliana*)
UGlcAe (1–6) At4g30440 At1g02000 At4g00110 At2g45310 At4g12250 At3g23820	D-UDP-GalA	Cytosol/Golgi	UDP-GlcA epimerase	Gu and Bar-Peled (2004) (*Arabidopsis thaliana*)
UGD (1–4) At1g26570 At3g29360 At5g15490 At5g39320	UDP D-GlcA	Cytosol	UDP-Glc dehydrogenase	Klinghammer and Tenhaken (2007) and Reboul et al. 2011) (*Arabidopsis thaliana*)
x	D-Dha	?	?	x
x	L-AceA	?	?	x

x = unidentified; ? = unknown.

### RG-II side chain and monosaccharide synthesis

Due to its complex structure, the synthesis of RG-II requires at least 21 specific glycosyltransferase activities that generate unique glycosidic linkages. To date, only a few enzymes needed for RG-II synthesis have been functionally characterized (Table 1). The first group, named RhamnoGalacturonan specific XylosylTransferases (RGXTs), are (1,3)-α-D-xylosyltransferases that catalyze the transfer on side chain A of xylose from UDP-α-D-xylose to *O*-methyl-α-L-fucose. These glycosyltransferases were localized in the Golgi by expression of GFP-tagged RGXTs and their colocalization with a Golgi marker (Egelund et al. 2006). The structure of the disaccharide obtained in biochemical assays performed using RGXT recombinant enzymes with UDP-α-D-xylose and methyl-α-L-fucose was confirmed by 1D and 2D ^1^H NMR (Egelund et al. 2006, 2008). Subsequently, another (1,3)-α-D-xylosyltransferase, MGP4 (or RGTX4), was identified and its mutation was demonstrated to impair pollen tube and root growth (Liu et al. 2011).

The second RG-II–specific glycosyltransferase, named Cdi, is a galactosyltransferase that was first studied in pollen, where its mutation caused a severe male gametophyte phenotype with lethality in homozygous plants (Voxeur et al. 2011; Li et al. 2012). In a recent paper, the rescue of plant fertility by expressing Cdi under a strong pollen promoter allowed the analysis of the phenotype in vegetative tissues. In this mutant line, root growth was strongly impaired concordantly with reduced RG-II dimerization (Peng et al. 2021). Mass spectrometry analyses showed a lack of L-galactose on RG-II side chain A, and the galactosyltransferase activity of Cdi was confirmed in vitro using a recombinant Cdi able to catalyze the transfer of L-galactose from GDP-L-galactose to RG-II fragments (Peng et al. 2021).

Other putative glycosyltransferases have been implicated in RG-II synthesis, but their activities have not yet been demonstrated in vitro. A glucuronosyltransferase (NpGUT1), expressed in *Nicotiana plumbaginifolia* callus, was proposed to function in the transfer of D-glucuronic acid to an L-fucose residue in RG-II side chain A. No in vitro activity assay was performed, but the mutant line lacked glucuronic acid in RG-II fractions and the dimerization rate was strongly decreased by comparison to wild type (Iwai et al. 2002). However, a putative ortholog of NpGUT1 in Arabidopsis, IRREGULAR XYLEM10-LIKE (IRX10-L), is instead hypothesized to function in xylan synthesis (Wu et al. 2009). A putative Kdo transferase was localized to the mitochondria and mutants for this gene showed no RG-II defects or morphological defects. Therefore, the authors suggested that Kdo transferase functions the synthesis of an unidentified mitochondrial lipid A-like molecule rather than in the synthesis of RG-II (Séveno et al. 2010). A CMP-KDO synthase (CKS) was also found in the mitochondria and mutants showed male gametophyte defect. However, the impact of the *cks* mutation on RG-II structure was only speculated because of the difficulties of getting RG-II from pollen culture (Kobayashi et al. 2011). Kdo and Dha are structurally related to sialic acid, which is found in animals but is absent in plants. Sialyltransferase orthologs have been found in plants, but they cannot use sialic acid as a substrate (Daskalova et al. 2009). Sialyltransferase-like proteins in plants have been postulated as candidates for the transfer of Kdo or Dha to RG-II side chains C and D, respectively (Séveno et al. 2010; Voxeur et al. 2012). Homozygous mutant lines for 2 sialyltransferase-like genes (*mgp2* and *sia2*) could not be obtained, and heterozygous lines exhibited severe defects in the germination and elongation of pollen tubes (Deng et al. 2010; Dumont et al. 2014). More recently, homozygous *mgp2* (or *rckt1-21*) callus was obtained via CRISPR/Cas9-genome editing, and the loss of function of this gene was studied (Zhang et al. 2024). RG-II isolated from *rckt1-21* callus walls by endoPG treatment of SEC fractions was present mostly in monomeric form (74%), whereas RG-II monomer accounted for 20% of the total RG-II in wild-type walls (Zhang et al. 2024). Both glycosyl composition and NMR spectroscopy showed the absence of Kdo in RG-II in *rckt1-21* callus (Zhang et al. 2024). Interestingly, Dha was detected in the *rckt1-21* mutant, implying that RCKT1 does not function as a Dha transferase. Altogether, the plant sialyltransferase-like *mgp2*/*rckt1* is likely a Kdo transferase required specifically for the transfer of Kdo to RG-II side chain C, although bioassays are needed for confirmation.

Furthermore, mutant lines related to the biosynthesis of cytosolic RG-II monosaccharides have been described, showing severe phenotypes (O’Neill et al. 2001; Ahn et al. 2006; Voxeur et al. 2011; Pabst et al. 2013; Qi et al. 2017; Zhao et al. 2020) (Table 2). Additionally, RG-II–specific nucleotide sugar transporters, such as the Golgi GDP-L-Galactose transporter 1, were reported (Sechet et al. 2018). Also, methylation and acetylation can occur in RG-II, but no RG-II–specific *O*-methyltransferases or *O*-acetyltransferases have been identified to date. However, the SAM transporters GOSAMT1 and GOSAMT2 have been proposed to be required for RG-II methyl-esterification by Temple et al. (2022).

### Approaches to decipher RG-II synthesis

The investigation of RG-II domain synthesis is a colossal challenge due to its complexity and importance in plant growth. To decipher RG-II biosynthesis, 3 main barricades need to be breached: (1) identify all the putative enzymes needed for RG-II synthesis; (2) circumvent potential issues of gene redundancy and plant lethality for loss of function mutants; and (3) confirm the activities of the glycosyltransferases in vitro in the presence of their native substrate and acceptor.

All the enzymes that have been found to function in RG-II synthesis thus far have been identified by either forward genetic or reverse genetic. With forward genetic approaches, screening for growth defects and sterility helped to discover several putative glycosyltransferases. For several mutant lines, growth defects could be partially restored by adding extra boric acid (O’Neill et al. 2001; Liu et al. 2011; Voxeur et al. 2011; Shi et al. 2017; Sechet et al. 2018), supporting the hypothesis that RG-II was disrupted. The other approach is reverse genetics following bioinformatic analyses such as levels of coexpression with genes already identified as related to RG-II or computational structure-function predictions as achieved in Voxeur et al. (2011). As the number of experimentally characterized enzymes increases, computational approaches will be even more powerful in uncovering additional putative enzymes for RG-II synthesis.

Genetic redundancy can prevent phenotypes from appearing in mutants lacking single genes involved in RG-II synthesis due to compensation by genes with similar functions. This redundancy can be overcome by generating multi-mutant lines by either crossing or multi-target CRISPR/Cas9 editing (Zhao et al. 2020; Zhang et al. 2024). Lethality prevents the propagation of homozygous lines with abnormal RG-II synthesis or dimerization, making it difficult to test for growth phenotypes in single-copy enzymes required for RG-II synthesis. In the case of a male gametophyte defect, expressing the cognate gene under a strong pollen promoter to bypass sterility is a valuable strategy to investigate phenotypes in vegetative tissues (Peng et al. 2021). Another innovative method is to perform CRISPR/Cas9-genome editing in callus and isolate cell walls from this tissue. In this way, RG-II defects that would normally cause sterility or lethality can be studied *in muro* to identify transferases and other proteins (Zhang et al. 2024). These strategies will help increase the number of characterized genes related to RG-II synthesis.

After the genetic identification of putative transferases, confirming enzyme activity in vitro is crucial. To confirm the function of a transferase, heterologous expression is needed with the purification of the target enzyme (Egelund et al. 2006, 2008; Liu et al. 2011; Peng et al. 2021). In vitro, the putative transferase must react with the cognate nucleotide sugar and transfer that sugar to an acceptor. This can now be done more quickly using higher-throughput screening approaches (Bhattarai et al. 2024). In the case of RG-II, because of its structural complexity, isolating a proper acceptor can be a daunting challenge (Pabst et al. 2013; Amos and Mohnen 2019). Chemoenzymatic approaches have been successful in synthesizing RG-II side chain fragments, which can be used as acceptors for enzymes, but only for side chain A and B so far (Rao and boons 2007; Rao et al. 2008; Lei et al. 2022). It is also possible to purify RG-II from mutant lines lacking a particular glycan, incubating it with the putative recombinant enzyme and its compatible nucleotide sugar. Mass spectrometry and NMR spectroscopy analysis can then be used to determine if the missing glycan motif has been transferred to the substrate (Peng et al. 2021). Lastly, a tremendous breakthrough was made by Ndeh et al. (2017) with the characterization of hydrolytic enzymes in gut bacteria, capable of cleaving most of the linkages in RG-II. These enzymes could be used to generate RG-II glycoforms suitable as acceptors for transferase assays in vitro (Amos and Mohnen 2019; Zhang et al. 2024). Altogether, the advancements made in the past 10 years have made great strides in decoding RG-II biosynthesis, providing new information and laying the foundation for further discoveries.

## RG-II detection in plant taxa, tissues, and cell walls

### RG-II in the plant kingdom and tissues

**Figure 2.**
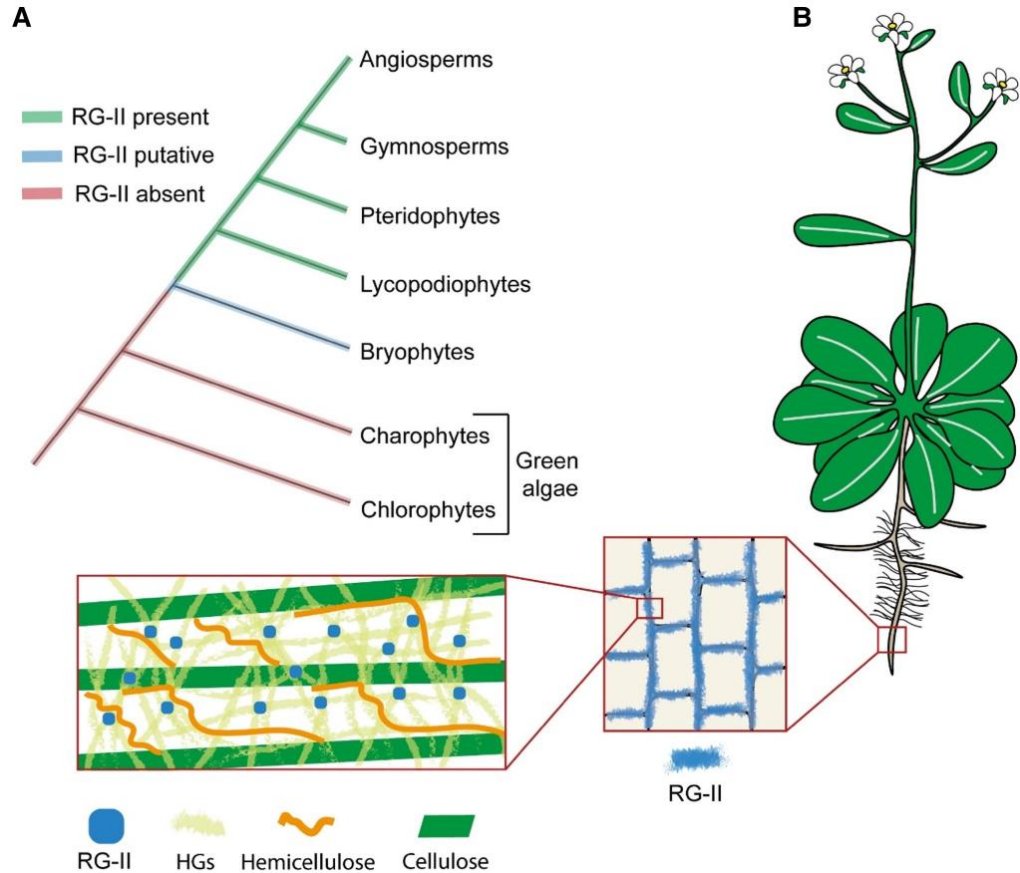
Localization of RG-II domain in plant taxa, angiosperm tissues and the cell wall. **A)** RG-II is present in terrestrial plants and absent in green algae; it is putatively present in Bryophyte species but has not been extensively studied in these taxa. **B)** RG-II is widely distributed in angiosperm tissues as detected by chemical analysis and microscopy observations. First inset: at the cellular scale RG-II is relatively evenly distributed as seen in microscopy analyses. Second inset: hypothetical distribution of RG-II at the nanoscale, based on the digestion of the pectin matrix by endopolygalacturonases. Schematics are not drawn to exact scale.

Data concerning RG-II in non-angiosperm plants is rare because these groups are understudied and obtaining material can be challenging. To date, RG-II has not been found in algae, so it seems that RG-II appeared during the terrestrialization of plants (Yin et al. 2009; Popper and Tuohy 2010; Begum and Fry 2023) (Fig. 2). Monocotyledons from the Lemnoideae subfamily, with an aquatic growth habit, conserved the presence of RG-II comparable to dicotyledons (Avci et al. 2018). In bryophytes (mosses, liverworts, and hornworts) only trace amounts of residues characteristic of RG-II were detected by GC-MS-EI in the alditol acetate derivatives, suggesting a lower quantity of RG-II in their walls (Matsunaga et al. 2004) (Fig. 2A). The amount of borate attached to RG-II was measured by SEC/ICP-MS and supports its presence in Bryophytes but in a lower amount compared with other land plants. The difficulty in obtaining tissues for chemical analysis is a limiting factor in the study of these organisms (Matsunaga et al. 2004). Further studies are required to confirm the presence of RG-II in bryophytes and describe the specificities of their structure. In gymnosperms and angiosperms, in tissues containing little boron, most of the existing boron is found in the cell wall in the form of crosslinked RG-II (Matoh et al. 1996; Shimokawa et al. 1999) (Fig. 2A). Also, the ability of RG-II to form a dimer was shown to be similar between all pteridophytes (Matsunaga et al. 2004). This can be explained by the high conservation of its structure among all these groups. While RG-II structure is often thought to be rigidly conserved, a few intraspecies and interspecies differences have been identified, with no effect on its dimerization capacity. Glycosyl composition and linkage analyses were performed on fractions of RG-II isolated by SEC. On the RG-II side chain B, in lycophytes and pteridophytes, 3-*O*-a methyl rhamnosyl residue linked to *O*-2 or *O*-3 to an arabinopyranosyl residue was detected, which is absent in angiosperms and gymnosperms (Matsunaga et al. 2004) (Fig. 1B). Few variations of methyl-esterification and arabinosylation were detected in plants from the Lemnoideae subfamily (Avci et al. 2018). Moreover, on RG-II side chain A, in wild types of plants from different bryophyte groups, distinct proportion of substitution of L-fucose by L-galactose were observed (Pabst et al. 2013). Together, this suggests a flexibility of RG-II structure in the plant kingdom, to potentially modulate its function in different species and/or organs. The occurrence of RG-II in all terrestrial plants, despite the cost of its complex synthesis, emphasizes its importance in the wall (Matoh et al. 1996; Pérez et al. 2003; Matsunaga et al. 2004; Pabst et al. 2013; Avci et al. 2018).

In angiosperms, RG-II has been detected in a variety of organs, tissues, and cells (Fig. 2B). RG-II is likely present in small amounts in seed mucilage in Brassicaceae, as detected by immunolocalization with an RG-II polyclonal antibody (Matoh et al. 1998) and biochemical detection of RG-II–specific sugars (Shi et al. 2017). Immunolabeling with polyclonal and monoclonal antibodies targeting RG-II (Baluška et al. 2002; Barany et al. 2010; Zhou et al. 2018; Hiroguchi et al. 2021), biochemical analyses (Matsunaga and Ishii 2006; Miwa et al. 2013; Muszyński et al. 2015), and metabolic labeling experiments (Dumont et al. 2016; Ropitaux et al. 2022; Hays et al. 2024) indicates the presence of RG-II in roots, including in root hairs. In the shoot vegetative parts of plants, because of autofluorescence, chemical analyses have been preferred over microscopy studies for the detection of RG-II (Voxeur et al. 2017). Several studies have used leaves to investigate RG-II, allowing the acquisition of a higher amount of material compared with roots (O’Neill et al. 2001; Reuhs et al. 2004; Matsunaga and Ishii 2006).

In reproductive organs, the presence of RG-II is suggested by the reduction in fertility and fruit development through RG-II related mutant lines or boron deficiency (Iwai et al. 2006; Liu et al. 2011; Mounet-Gilbert et al. 2016; Qi et al. 2017). In pollen tubes, an invasive fast-growing cell crucial for pollination, more evidence is available. Mutant lines related to RG-II dimerization or synthesis displayed growth defects in pollen tubes (Delmas et al. 2008; Deng et al. 2010; Liu et al. 2011; Dumont et al. 2014; Qi et al. 2017; Zhao et al. 2020), and immunolabeling and metabolic labeling allowed the observation of RG-II in the cell walls of pollen tubes (Dumont et al. 2014; Ropitaux et al. 2022). Additionally, Kdo was detected in pollen cell walls, suggesting the presence of RG-II (Dumont et al. 2014).

### RG-II in the plant cell wall

At the molecular scale, RG-II domain is localized in the pectin matrix (Fig. 2B), but details of its interactions with other polysaccharides are still mostly unknown. It is thought that the pectic domains HG and RG-II, and HG and RG-I, are linked covalently to each other, suggested by data obtained from chemical and enzymatic degradation experiments (Ishii and Matsunaga 2001; Vincken et al. 2003; Mohnen 2008; Delmer et al. 2024). Specific fractions of pectins are also thought to associate closely with other constituents such as cellulose (Wang et al. 2012, 2015; Phyo et al. 2017). Whether RG-II is important for pectin-cellulose interaction is not known, but evidence points to RG-I side chains (arabinan and galactan) as main actors, with HGs playing smaller roles (Zykwinska et al. 2007; Wang et al. 2012, 2015). Recently, it was found that the main form of arabinogalactan proteins extracted from different tissues in *Arabidopsis thaliana* were pectic-AGPs (Tan et al. 2024). Moreover, AGPs have been proposed as natural cationic chaperones in the Golgi apparatus and/or in the apoplast to promote RG-II dimerization (Sanhueza et al. 2022). Some data support direct bonding between pectins and hemicelluloses (Popper and Fry 2008). However, because they are both synthesized in the Golgi apparatus and potentially transported in the same vesicles, inherent proximity between these polysaccharides could also be hypothesized (Popper and Fry 2008). Altogether, RG-I and HGs are currently better candidates than RG-II for proximity with celluloses and hemicelluloses. RG-II domain is thought to be mainly localized in the pectin matrix, interacting with HGs and itself (Fig. 2B).

### New tools for RG-II imaging in plant cells

RG-II has been detected in plants mainly through chemical analyses after extraction and purification of the polysaccharide. Cell imaging using a polyclonal antibody raised against RG-II (Matoh et al. 1998) has also been performed (Matoh et al. 2000; Baluška et al. 2002; Kasprowicz et al. 2009; Barany et al. 2010; Dumont et al. 2014; Shi et al. 2017; Zhou et al. 2017), but clear patterns of subcellular localization were not observed. However, this antibody is not currently available, leading to a lack of information about RG-II localization *in muro* compared with other polysaccharides. A monoclonal antibody was recently made, recognizing RG-II monomer and dimer but also an unknown epitope (Zhou et al. 2018; Hiroguchi et al. 2021). Doubts about its specificity make it difficult to use, so metabolic labeling, consisting of the application of a modified sugar that can be absorbed by the cell and incorporated in the target molecule through cell machinery, has been developed as an alternative labeling method. This modified sub-unit contains a non-native chemical functionality that can be covalently bound to a complementary moiety linked to a fluorescent probe through a biorthogonal reaction (Rydahl et al. 2018). Metabolic labeling is used to detect the incorporation of exogenously added sugars into animal and bacterial cells and has been developed for plants in the past 10 years (Anderson et al. 2012; Hoogenboom et al. 2016; Zhu and Chen 2017). Due to the unique sugars found in RG-II, this method provides a promising alternative to antibodies for localizing RG-II (Dumont et al. 2016; Ropitaux et al. 2022; Hays et al. 2024). For example, metabolic click-labeling of RG-II can be performed by feeding a plant with a Kdo analog. After conversion to a nucleotide sugar via the salvage pathway, transfer of the Kdo analog to RG-II, and delivery to the wall, the chemical tag can be covalently linked to a fluorescent probe, for example, by a bio-orthogonal azide-alkyne cycloaddition reaction (Dumont et al. 2016; Ropitaux et al. 2022). Metabolic click labeling of RG-II was first carried out using copper as a catalyst. This metal ion is toxic for plant cells at the concentration needed to catalyze the click-reaction (Anderson et al. 2012; Dumont et al. 2016). A copper-free click chemistry reaction was then adopted and allowed for live imaging of fast-growing plant cells such as root hairs and pollen tubes (DeVree et al. 2021; Herburger et al. 2022; Ropitaux et al. 2022). Together, the development of new antibodies and metabolic labeling along with super-resolution and/or electron microscopies might provide a new window into the nanoscale distribution of RG-II domain in the cell wall.

## RG-II dimerization and function

### When and where does RG-II dimerize?

**Figure 3.**
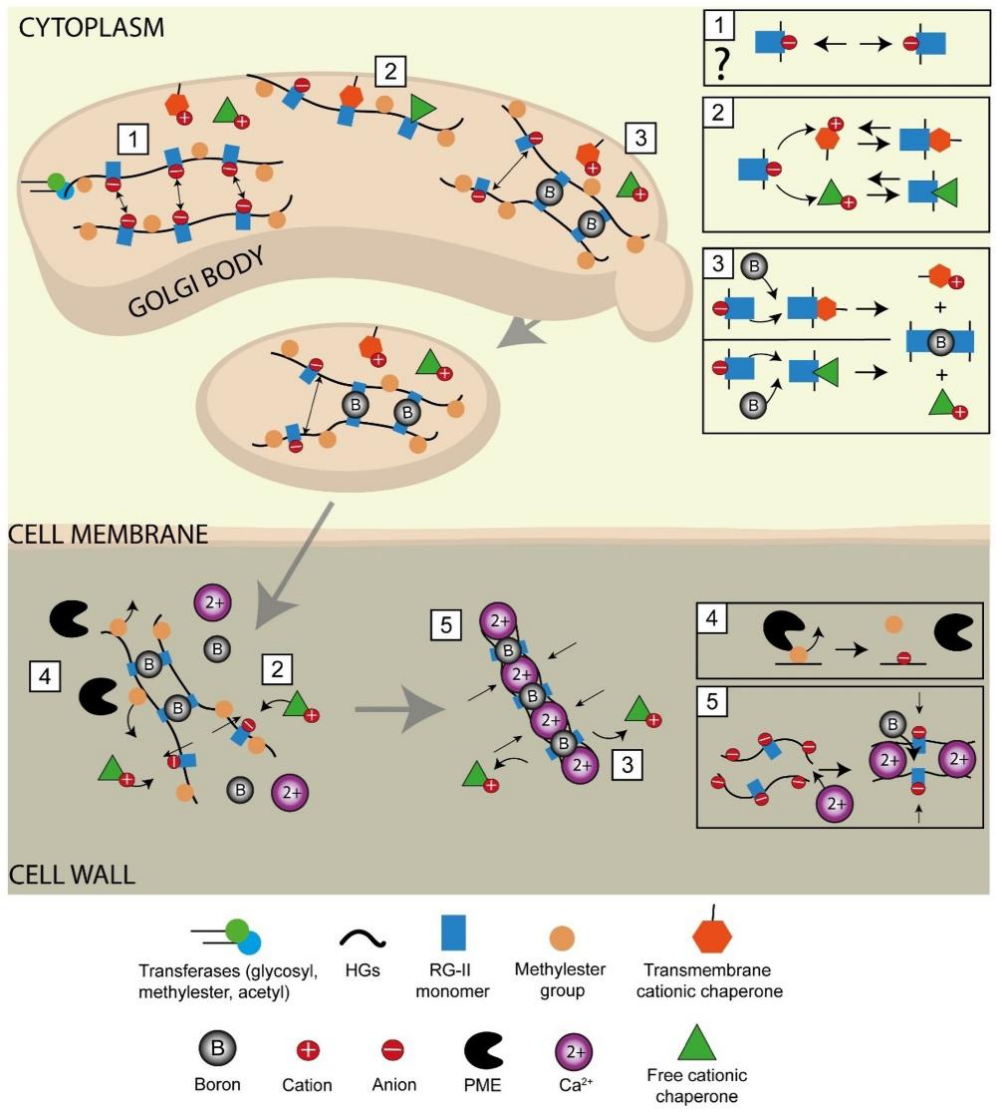
Potential mechanisms for RG-II dimerization. After being synthesized in the Golgi, RG-II monomers and the HGs to which they are bound are negatively charged and might repel RG-II from other pectin chains, preventing crosslinking **(1)**. Question mark highlights uncertainty regarding the degree of methyl-esterification for the RG-II backbone (O’Neill et al., 2020). Proteins with cationic domains might act as chaperones with RG-II monomers and neutralize their charges (Chormova and Fry, 2016) **(2)**, removing the repulsion force and allowing 2 monomers to be close enough to dimerize with boron **(3)**. When delivered to the cell wall, the methyl-ester groups on HGs can be removed by PMEs **(4)**. Negatively charged HG chains can be crosslink with calcium ions, which could create favorable conditions for RG-II monomers to crosslink **(5).**

In plant tissues containing low boron, more than 90% of boron is found in the cell wall (Hu and Brown 1994; Matoh et al. 1996), implying that RG-II might be bound to much of this boron. In the pectin matrix, RG-II is mainly present as a dimer through a borate diester bond (Matsunaga et al. 2004). Polyacrylamide electrophoresis gels have been extensively used to study RG-II dimerization (Chormova et al. 2014; Voxeur and Fry 2014; Chormova and Fry 2016; Sanhueza et al. 2022; Begum and Fry 2023; Begum et al. 2023). This technique allowed the separation of RG-II monomer and dimer on a gel and the semi-quantitative determination of their proportion. Interestingly, despite evidence showing that only the apiose residue from side chain A is involved in RG-II dimerization (Ishii and Matsunaga 1996; O’Neill et al. 1996; Pellerin et al. 1996; Ishii and Ono 1999; Ishii et al. 1999), unstable RG-II trimers and tetramers have been observed in these gels, suggesting a potential interaction between apiose from side chain B and boron (Begum and Fry 2023). Although a lack of boron is lethal in most cultured plant cells, suspension cultures of *rosa* cells can be grown in boron-free media and used as a model to investigate RG-II crosslinking (Chormova et al. 2014). In boron-free media, these cells produce walls containing mainly RG-II monomer with very few dimers detected (Chormova et al. 2014; Sanhueza et al. 2022; Begum et al. 2023). Supplementation with boric acid led to the observation of RG-II dimers only after 24 h, suggesting that the dimerized RG-II was mostly newly synthesized rather than already being present in the wall (Chormova et al. 2014). More recently, RG-II monomer and dimer synthesis were tracked by pulse labeling experiments with ^14^C glucose in plants fed with glycerol as a carbon source. ^14^C was detected in both monomers and dimers and its incorporation occurred at a linear rate, confirming de novo ^14^C-RG-II synthesis (Begum and Fry 2022). RG-II dimer containing ^14^C was observed within 4 min of incubation, while the travel time from the Golgi to cell wall is about 15 to 20 min, implying that this RG-II dimer is mainly intracellular (Begum and Fry 2022) (Fig. 3). However, after ^14^C glucose washout, the ^14^C RG-II dimer continued to increase while the monomer stayed mostly unchanged. Thus, this result raised the possibility that RG-II dimerizes post-secretion in the apoplast (Begum and Fry 2022) (Fig. 3). Moreover, in *Chenopodium album* and *Cucurbia moschata* cultured in boron-free medium, supplementation with boric acid led to a high rate of dimerization, which could suggest that some RG-II dimerization occurs in the apoplast (Fleischer et al. 1998; Ishii et al. 2001). Altogether, RG-II is likely to dimerize mainly in the Golgi, but we hypothesize that the proportion of RG-II that dimerizes in the apoplast might be underestimated. After incorporation into the pectin matrix, favorable physicochemical conditions for crosslinking might be established, gradually leading to the formation of boron bridges in the apoplast and a higher rate of RG-II dimerization (Fig. 3).

### Mechanisms and actors behind RG-II dimerization

While the dimerization rate of RG-II is high in vivo, in vitro experiments have shown that RG-II and boric acid alone dimerize poorly (Sanhueza et al. 2022). It has been hypothesized that because RG-II is negatively charged, electrostatic repulsion prevents the proximity needed to form the boron bridge (Chormova and Fry 2016; Sanhueza et al. 2022) (Fig. 3). In vitro, addition of divalent metal ions or cationic chaperones increases the RG-II dimerization rate (Chormova and Fry 2016; Sanhueza et al. 2022; Begum et al. 2023). We hypothesize that in vivo, cationic chaperones such as extensins might neutralize RG-II and allow 2 RG-II-containing molecules to come close enough to form a borate diester bridge both in the Golgi vesicles and the apoplast (Chormova and Fry 2016) (Fig. 3). However, even at a pH where RG-II is neutral, RG-II monomers and boric acid in the absence of cationic agents do not lead to an increase in the crosslinking rate (Begum et al. 2023). This result indicates that the function of cationic chaperones might not be simply to neutralize RG-II but include something more complex (Begum et al. 2023). In vitro, a low concentration of Pb^2+^ and high concentration of Ca^2+^ facilitate the dimerization of isolated RG-II monomers at acidic pH via coordinate covalent bonds (Begum et al. 2023). Also, glycoproteins with cationic domains such as polyhistidine, expansins or arabinogalactan proteins (AGPs) are candidates as cationic chaperones to facilitate RG-II dimerization. These proteins, even at low concentration, improve the dimerization rate of isolated RG-II in vitro, supporting their putative chaperone function in vivo (Chormova and Fry 2016; Sanhueza et al. 2022; Begum and Fry 2023). Moreover, AGP 31, which increases RG-II crosslinking in vitro, interacts via ionic bonds with HGs and RG-I, potentially contributing to cell wall assembly (Hijazi et al. 2014). These data are useful to decipher the putative actors behind RG-II crosslinking. However, *in muro*, RG-II domains are not isolated but are covalently linked to HGs and exposed to different conditions than in vitro. If we consider that a portion of RG-II is dimerized before being secreted to the cell wall, upon its incorporation into the pectin matrix favorable conditions for crosslinking could be established, gradually making boron bridges in the apoplast, leading to a higher rate of RG-II dimerization over time (Fig. 3). In this context, cationic chaperones might initially prevent complete dimerization of RG-II until its delivery to the cell wall and then dissociate from the RG-II, letting boron-mediated dimerization occur. It is also conceivable that RG-II monomers from different pectin chains have little opportunity to be in contact because HGs are methylesterified and are thus incapable of crosslinking before being sent to the apoplast (Fig. 3). When PMEs remove methylester groups and HGs form calcium-mediated egg box structures, closer proximity between different pectin chains might evolve, allowing RG-II monomers to be close enough to crosslink (Fig. 3). High concentrations of boric acid could also promote RG-II dimerization in the apoplast. This could explain why some RG-II related mutants show partially restored dimerization when supplemented with high amounts of boric acid (Fig. 3) (O’Neill et al. 2001; Liu et al. 2011; Voxeur et al. 2011; Peng et al. 2021). However, how boric acid increases crosslinking in these mutant lines is still unknown but does not seem to occur in all RG-II mutant lines (Zhao et al. 2020).

### RG-II structure conditions its dimerization

RG-II structure is highly conserved in the plant kingdom (Matsunaga et al. 2004), suggesting a structure-function relationship. As discussed above, RG-II is mainly found as a dimer in the wall. Mutant lines related to RG-II defects display incomplete RG-II structures, leading to a decrease in the dimerization rate (O’Neill et al. 2001; Liu et al. 2011; Pabst et al. 2013; Voxeur et al. 2011; Qi et al. 2017; Peng et al. 2021; Zhang et al. 2024). On side chain A, the absence of 2-*O*-MeXyl (Liu et al. 2011), L-Gal (Peng et al. 2021), or D-GlcA (Iwai et al. 2002) prevents RG-II crosslinking, underlining the importance of this side chain for dimerization. In the *Arabidopsis thaliana* mutant line *mur1*, RG-II side chain A L-Fuc is replaced by L-Gal (O’Neill et al. 2001; Reuhs et al. 2004; Pabst et al. 2013). Interestingly, in some species, this replacement is naturally present without impairing the ability of these plants to form dimers (Pabst et al. 2013). It was then hypothesized that it is not the substitution of L-Fuc/L-Gal itself that causes reduced dimerization in *mur1* but the truncation of the side chain (Pabst et al. 2013). Pharmacological treatment using peracetylated 2F-Fuc also disrupts RG-II side chain A formation by inhibiting RG-II fucosylation in the Golgi (Dumont et al. 2015; Carlier et al. 2023). On the other hand, the absence of Kdo-containing side chain C in the *rckt1-21* mutant line leads to a diminution in the ability of RG-II to form dimers (Zhang et al. 2024). Moreover, treatments with 2β-deoxy Kdo, which inhibit CMP-Kdo-synthetase, strongly impair seedling growth, suggesting a defect in RG-II dimerization, synthesis, and/or deposition (Smyth et al. 2013; Dumont et al. 2016). Altogether, these results suggest that side chain C is needed for RG-II to properly dimerize. For side chain B, variation in RG-II structure between angiosperm plant tissue, wine, and Pteridophytes can be observed (Matsunaga et al. 2004; Pabst et al. 2013; Ndeh et al. 2017). The additional monosaccharides (*O*-methyl-L-Rha, L-Ara) and modifying groups (*O*-Me and *O*-Ac) found in wine or Pteridophytes might not be needed for the dimerization, as suggested by their absence in angiosperms (Matsunaga et al. 2004; Pabst et al. 2013; Ndeh et al. 2017). To date, no conclusive data supports the idea that side chain D is required for RG-II dimerization. However, it is possible that as for Kdo, Dha is involved in RG-II dimerization and that a bioassay with SIA2, a putative Dha-transferase, could provide additional insight once purified Dha and/or its nucleotide form are available (Dumont et al. 2014). The breakthrough made by Ndeh et al. (2017) with the enzymatic digestion of RG-II by gut bacteria will enable the generation of an RG-II glycoform library. Having a diversity of RG-II substrates for in vitro assays will help to decipher the fine balance between structure and the dimerization. It has also been shown that in mutant lines with RG-II defects, crosslinking could be restored by high concentrations of boric acid (O’Neill et al. 2001; Liu et al. 2011; Voxeur et al. 2011; Peng et al. 2021). How this excess of boron interacts with defective RG-II and enhances its dimerization is still unknown, but could be due to the presence of more favorable crosslinking conditions in the apoplast (Fig. 3).

### RG-II dimerization is required for its functions in plants

Despite representing 0.5% to 5% of the cell wall content (Darvill et al. 1978; Matsunaga et al. 2004; Barnes et al. 2021; Lerouge et al. 2021), RG-II domain is crucial for maintaining the cell wall properties required for plant growth, development, and reproduction. Although absolute quantification of RG-II content is difficult due to its low abundance in the cell wall, measuring RG-II dimerization provides a diagnostic tool in mutant lines or after pharmacological treatments (O’Neill et al. 2001; Liu et al. 2011; Voxeur et al. 2011; Dumont et al. 2015; Sechet et al. 2018; Zhao et al. 2020; Peng et al. 2021). Existing data strongly suggest that RG-II crosslinking in the cell wall is required for its function while RG-II monomer alone does not seem to contribute to cell wall strengthening, cohesion, or adhesion. This idea is also supported by boron deprivation experiments that induce similar effects as mutations or pharmacological treatments, even if boron is thought to have functions other than interacting with RG-II (Miwa et al. 2013; Bolaños et al. 2023). Another strategy for probing the effects of RG-II dimerization is the use of analogs of boron such as phenylboronic acid (PBA), which have been hypothesized to compete with boron for binding to monomeric RG-II, preventing crosslinking (Bassil et al. 2004; Matthes et al. 2020). In fact, PBA binds to RG-II but does not inhibit its dimerization, and even seems to allow the formation of RG-II dimers in the absence of boric acid in vivo (Hays et al. 2024). Additionally, PBA does not only interact with RG-II but also has anti-auxin effect (Hays et al. 2024). A recent study has shown that in *Arabidopsis thaliana mur1* mutant line with RG-II dimerization defects, the expression of auxin response factors and brassinosteroid biosynthesis genes were downregulated, inducing defective apical hook development (Jewaria et al. 2025). Interestingly, the recovery of auxin response factors expression of and brassinosteroid biosynthesis restored the apical hook development in *mur1* and also the brassinosteroid could enhance RG-II dimerization (Jewaria et al. 2025). These results highlight that in the case of an impairment in the cell wall integrity induced by a defect in RG-II dimerization, hormones can influence the wall to partially restore RG-II crosslinking and maintain growth. Altogether, these results indicate that RG-II must be dimerized to function properly in plants.

**Figure 4.**
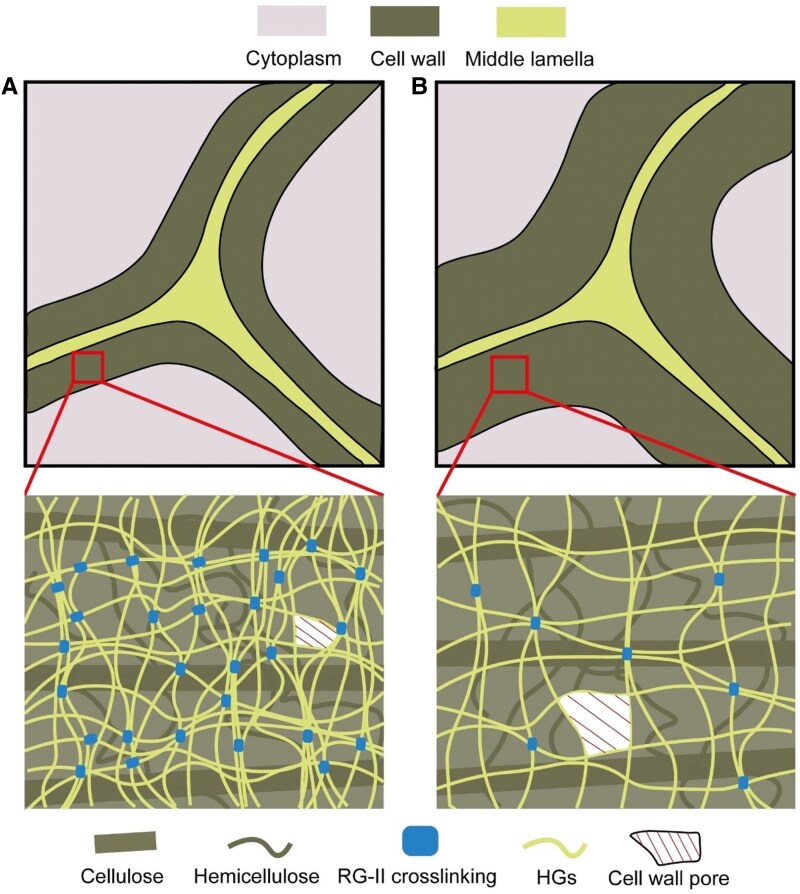
Functions of RG-II in determining cell wall properties. **A)** Wild-type cell wall containing mainly RG-II crosslinked within a dense pectin matrix. **B)** Mutant line with defects in RG-II dimerization, depicting a less dense pectin matrix with an increase in cell wall thickness and porosity. Schematics are not drawn to exact scale.

At the cellular scale, 1 symptom of impairment in RG-II crosslinking is cell wall swelling (Ishii et al. 2001; Ahn et al. 2006; Biswal et al. 2018; Zhao et al. 2020) (Fig. 4). Potentially as a consequence of this swelling, cell wall porosity is also higher (Fleischer et al. 1998; Biswal et al. 2018; Liu et al. 2021) (Fig. 4), suggesting a reduction in the density of the pectin matrix. This reduction in density disrupts the physical properties and integrity of the cell wall (Ryden et al. 2003; Shi et al. 2017; Panter et al. 2023). A major function of RG-II is likely to densify and maintain the correct porosity of the cell wall through its crosslinking with boron, which might explain why RG-II monomers do not seem to contribute to cell wall properties (Fleischer et al. 1998; Biswal et al. 2018; Liu et al. 2021; Obomighie et al. 2025). Wall loosening and increased porosity coincide with numerous developmental and physiological phenotypes in plants with defective RG-II. For example, multiple mutant lines related to RG-II are sterile, displaying impaired pollen growth through the style (Delmas et al. 2008; Deng et al. 2010; Dumont et al. 2014). Mutant lines and plants treated with pharmacological agents (2F-Fuc or 2β-deoxy Kdo) or lacking boron show dwarf phenotypes with a disruption of cell expansion and cell organization (O’Neill et al. 2001; Liu et al. 2011, 2021; Peng et al. 2021). Cell-cell adhesion can also be impaired when RG-II is not properly dimerized (Iwai et al. 2002; Bassil et al. 2004). Suspension cultures of *Rosa* cells can be adapted to B deficiency and show little RG-II dimerization (Chormova et al. 2014), but these cultures likely do not require the cell adhesion or tissue mechanics needed by intact plants. Both *mur1* and B deficiency in Arabidopsis hypocotyls displayed a lower tensile strength, which can be rescued by boron supplementation, supporting a potential function for RG-II in maintaining the mechanical strength of the cell wall (Hu and Brown 1994; Fleischer et al. 1998; Ishii et al. 2001; Ryden et al. 2003). Additionally, higher wall porosity correlates with an increase in cell wall extractability in *gaut4* mutant lines in switchgrass and poplar (Biswal et al. 2018) and in seed mucilage in the *mur1* mutant of *Arabidopsis thaliana* (Shi et al. 2017), implying a loss of structural cohesion in the cell wall. The more porous cell wall in RG-II mutant lines is apparently less resistant to abiotic stresses such as freezing and drought compared with wild type (Panter et al. 2019; Liu et al. 2021). Moreover, severe water loss and diminished leaf conductance are observed in *mur1* plants and can be partially rescued by supplementation with boric acid, implicating RG-II in water management (Panter et al. 2023; Waszczak et al. 2024). Taken together, the densification of the macromolecular pectin matrix by RG-II crosslinking is crucial to maintain cell wall integrity and properties for cell expansion, cell adhesion, stomatal conductance, and the ability to endure abiotic stresses.

### Questions, hypotheses, and avenues for future research

Many unanswered questions remain concerning RG-II structure, taxonomic distribution, synthesis, trafficking, dimerization, location in the wall, and function. From an evolutionary point of view, it is interesting to note that the 3D structure of side chain A is conformationally optimized to facilitate a high affinity of the apiose residue for the borate and subsequent dimerization. As this is not the case for the apiose residue of side chain B, it raises the question of the appearance of this structural innovation since it is generally accepted that RG-II originated from a common ancestor of all plants. In particular, one can ask whether this common ancestor already contained RG-II domain and if side chain A arose from a precursor glycan sequence that unexpectedly acquired a high affinity for environmental borate, leading to a “gain of function” for the primary cell wall. It is even possible that this glycan-boron interaction was acquired via horizontal gene transfer from another taxon. Answers might come from the study of RG-II domain in grasses, which contain small amounts of pectin compared with eudicots, and also from the study of RG-II structure and dimerization capacity from a wide range of plant taxa.

In a general manner, the synthesis of pectin is still a matter of debate, as the precise location within the Golgi apparatus and the sequence of action of the enzymes involved in pectin biosynthesis is still unknown. A recent paper reporting gene editing of the Kdo-T (Zhang *et al*. 2024) showed that depletion of Kdo-transferase affected RG-II dimerization even though the Kdo-containing side chain C is not involved in boron-mediated dimerization. This result raises the possibility of the existence of a quality control step for the completion of RG-II synthesis before its dimerization and/or an RG-II integrity sensing mechanism that might depend on the successful synthesis of side chains C and D. Transcriptome profiling of B-deprived or RG-II mutant plants, suppressor screens with RG-II mutants and the development of new powerful imaging techniques such as Correlative Light Electron Microscopy together with specific labeling of RG-II via metabolic labeling will help answer these questions in the future. Correlative Light-Electron Microscopy should allow researchers to simultaneously detect pectin-synthesizing enzymes and their polysaccharide product in the Golgi apparatus and secretory vesicles at the nanoscale. This will help to address several fundamental questions regarding pectin and RG-II biosynthesis, in particular whether different pectic domains share the same secretory vesicles and whether the pectic content of the vesicles differs according to the zone of secretion. RG-II has been proposed by Dumont et al. (2015) to play a role in cell elongation and the development of these imaging techniques combined with structural analysis of RG-II from fast-growing cell types, such as pollen tube or root hairs, as well as biomechanical studies of RG-II–defective cells, might shed more light on this subject.

## Conclusion

RG-II domain is a widely conserved and enigmatic wall polymer but is beginning to yield up some of its secrets following advances in phylogenetic analysis, structure determination, genetic engineering, molecular phenotyping, and pharmacological manipulation. The ability to control RG-II synthesis, abundance, localization, dimerization, and degradation has the potential to allow researchers to tune the physical and mechanical properties of cell walls to enhance plant growth, stress resilience, and the utility of plants and plant-derived products for sustainable bioeconomy.

## Data Availability

No new data were generated or analysed in support of this research.

## Dive Curated Terms

The following phenotypic, genotypic, and functional terms are of significance to the work described in this paper:

GAUT1 Gramene: AT3G61130
GAUT1 Araport: AT3G61130

GAUT4 Gramene: AT5G47780
GAUT4 Araport: AT5G47780

GAUT7 Gramene: AT2G38650
GAUT7 Araport: AT2G38650

GAUT14 Gramene: AT5G15470
GAUT14 Araport: AT5G15470

GAUT11 Gramene: AT1G18580
GAUT11 Araport: AT1G18580

mur1 Gramene: AT3G51160
mur1 Araport: AT3G51160

mur4 Gramene: AT1G30620
mur4 Araport: AT1G30620

RGXT1 Gramene: AT4G01770
RGXT1 Araport: AT4G01770

RGXT2 Gramene: AT4G01750
RGXT2 Araport: AT4G01750

GAUT13 Gramene: AT3G01040
GAUT13 Araport: AT3G01040

GME Gramene: Acc04223
GME Araport: Acc04223
